# Pure Homœopathy, Progressive Homœopathy, and the True Homœopathician

**Published:** 1882-12

**Authors:** Daniel W. Clausen

**Affiliations:** Auburn, N. Y.


### PURE HOMŒOPATHY, PROGRESSIVE HOMŒOPATHY, AND THE TRUE HOMŒOPATHICIAN.

Daniel W. Clausen, M. D., Auburn, N. Y.

*Read before the "Central New York Homœopathic Society," at Syracuse, 15th of June, 1882.*

The word *Homœopathy* has but one literal signification; but, unfortunately, that signification does not restrict the word to its legitimate use. It is very much like the word *Christian*, which to-day means quite a different thing to what it meant when the disciples were first called Christians at Antioch. Indeed, one can hardly fail to see the striking similarity between the word *Homœopathy* and the parable of the "grain of mustard seed," which, as our Lord said in His comparison, "Though it be the smallest of all seeds, groweth to a large tree, so that the fowls of the air come and lodge in the branches thereof."

*Homœopathy*—the small seed sown in Germany nearly a century ago—is to-day a large tree; and under the cover of its branches are illegally lodged a great many birds of foreign flight, whose feathers, indeed beautiful in outward appearance, yet retain their tincture hues of yellow, red, and blue; while the cross-breeds are *ad infinitum;* and there is that imperious-looking rooster that seems to proclaim with every flap of his wings, "Liberty of action," and with every crow, "Freedom of opinion."

Even a kid ("Kidd") has been known to climb this tree, besmearing its tender branches with an innumerable quantity of his

Pillulæ capricorni ("goat pills") and disturbing the quietus of the peaceful doves with his most terrific "bah!"

One "Browne" (Dyce) once hung his clothes on the tree, and attempting to wash in the sparkling stream that nourishes the tree, found the water too deep for him, poor fellow! and so he nearly got drowned, like his namesake of the firm—"Smith, Brown, Jones, and Robinson"—of ancient story. His full recovery is doubtful.

But, with all the trespasses of these illegitimate refugees, besides exposure to frequent showers of hail ("Hale") from the northwest, the tree with all its foliage bears still the original impress imparted to it by the germ. Nothing can alter her truthfulness and faithfulness to nature; nor is even he that hews ("Hughes") able to destroy it with his ax, though he be considered a power in the field (of "Pharmacodynamics").

The various meanings applied to the word *Homœopathy* to-day make the title decidedly Hibernian in character, as Dr. Skinner would say; for it is used to mean: (1) Truth, (2) Error, and (3), what is worse, the harmonious co-existence of Truth and Error. This is hardly admissible in an age like the present, when people profess to be so much wiser than they who lived in the age of "the philosopher's stone."

Consequently, for the sake of distinction, the word *Homœopathy*, as understood by true homœopathicians, requires the use of an *adjective*, which is best met by the word *pure*. The adjective, "*legitimate*," may apply simply to the strict observance of the *law* of the similars, without necessarily including in its signification all or any of the *purities* that pertain to genuine homœopathic practice, such as the potency, dose, repetition, and a thousand other niceties.

Among those who have done most to corrupt the doctrine of pure Homœopathy are, notably, E. M. Hale, M. D., of Chicago, and Richard Hughes, M. D., L. R. C. P., of England, who, concerning the truth, have erred; whose attempts to convert Homœopathy into eclecticism and to adorn it with the brass-gold buttons of a "physiological and pathological livery" have by their respective works *ruined* a multitude of medical students—students who started with the honest intention of studying *pure* Homœopathy, but have been unfortunately caught in the snares and delusions of these eclectic and "pharmacodynamic" teachers.

Pure Homœopathy admits of no such thing as "the pathological sphere of action" of such or such a remedy. If we could say such is the pathological sphere of action of this or that drug our materia medica would lose its vast comprehensiveness and its study be reduced to mere "child's play." But a remedy is limited to *no* "pathological sphere of action;" far from it, the immense variety of phenomena presented in its symptomatology renders it applicable to an almost equal variety of diseases, whose respective "spheres of action" are totally different, and it is applicable in each of these diseases, just as any of these various phenomena (symptoms) in the sick furnish indications for its use. Belladonna is as much homœopathic to some cases of uterine disease as it was to the old Sydenham scarlatina. And where is the harmonious link in the pathology of the two diseases? Do they come within one "pathological sphere"?—are they of one family? Nay; but if any form of disease never before seen were to appear, the wide spheres of action of our remedies—not limited to any special "pathological spheres"—would render them applicable to such disease. Moreover, if a remedy were limited to any "pathological sphere of action" the ever-varying, ever-changing forms of disease would, in course of time, render the remedy comparatively, if not altogether, useless; instead of that, our oldest remedies are the indispensables of to-day.

Why will professors in "homœopathic" medical colleges so persistently endeavor to teach that a system of therapeutics, even of Homœopathy, must be based upon a knowledge of pathological changes? Such teaching is a departure from the true faith, and is neither more nor less than going back to the ante-Hahnemannian ages of *darkness* and *blind ignorance*—"*A fatal error*," as our much-loved Dr. Lippe would say.

Supposing even it were always possible for us to know what was going on in the hidden interior of man, this would not help us one mite as regards therapeutics. Pathological changes and processes are not disease, but *result from disease*, *i. e.*, from "a dynamic alteration of the vital force." As one, in a certain place, has truly said: "Living manifestations of disease are exact expressions of their internal nature, and organic lesions are consecutive results of the primary morbid activity of the vital force." And what do we understand by "living manifestations of disease"? For these we have not to go to the cadaver, nor do we understand them to be fully expressed by any visible pathological changes on the living subject, but they present themselves to us in a variety of phenomena called *symptoms*, speaking with the voice of nature; hence, "*living* manifestations"

—physiologically alive—the expressions of perverted physiological functions not yet dead—not in the "dead house."

The consecutive results of the primary morbid activity of the vital force are only *dead manifestations*. Hence it is that we get such excellent and wonderful results when we apply the dynamized —spiritualized—medicinal agent in harmony with the "*living manifestations*."

How naturally vast, then, must be the difference between treatment according to the deductions drawn from "dead-house pathology," and that according to the indications furnished by "living manifestations!" Oh! what a great and luxuriant tree is this tree of Homœopathy!—a tree whose leaves are for the healing of the nations; a tree that is not withered by the influence of autumn nor blighted by the cold blasts of winter, but has a perpetual existence, being watered with the refreshing dews of progressive Homœopathy from the hands of those noble veterans whose images stand depicted in the crystal drops as they lie clustered on every leaf.

Pathological changes are always *preceded* by *symptoms* of the disease; hence, it is necessary to prove our remedies only to the extent of eliciting certain characteristic symptoms, *never* to the extent of producing pathological changes—a statement that is substantiated by the fact that the remedies *do cure* pathological changes when selected according to symptomatic indications. I do not believe that *Aurum* was proved to the extent of producing caries of the palatine bones; and, certainly, *Belladonna* never produced the "Scarlatina."

When the late Dr. Carroll Dunham cured an ovarian tumor with Colocynth $^{200}$ he did not select his remedy as one that had ever been known to produce or to cure *that pathological condition;* he never thought of the disease *by name;* he endeavored to cure his *patient* by considering his *patient's constitutional symptoms;* and when the *patient* was cured the tumor disappeared, because it could not exist in a healthy organism. It was on the same principle that Hahnemann once cured a case of "fig-warts" with Chamomilla$^{30}$. Latter-day Homœopathy would teach to look for the rubric "fig-warts," and the prescriber, prescribing, of course, for the disease (*i. e.*, for the *name*) and not for the patient, would be confined to a choice between two or three remedies, and so—FAIL TO CURE.

Latter-day Homœopathy teaches to treat "worms" as a disease, and so to follow the careless routine of administering "anthelmintics" to every subject supposed to be infested with these parasites, and because the doctor is told that "lots of worms" have been passed he fondly prides himself in the imagination that his patient is cured, receiving a full share of commendation from all the old women who happen to catch a glimpse of "the vessel." But, after all, it may be that these worm-doctors evince a fair degree of acumen in trying to become popular among the women; for it is a remarkable fact that you cannot please certain women better than to make them believe that you are going to expel from them a *worm*, or a *snake*, or a *tumor*, or some other imaginary incumbrance. And right here I am reminded of a case in point, which occurred at a medical college while I was there attending a course of lectures. At the "gynecological clinic" there came a woman one day suffering from some uterine disorder, and, in addition to various phases of nervous mimicry, she fancied that she had a *snake* within her. The professor, a fairly keen gentleman, did not, of course, try to disabuse her of her belief, but aimed at the uterine trouble, resorting to his usual mode of treatment, which included the insertion of the tampon, or plug of cotton, well lubricated with vaseline and having a string attached for the purpose of withdrawal. She did not, however, know what was being done to her. In a day or two she called at the doctor's office, and, with concurrent expressions of great joy and absolute certainty, she exclaimed: "Hah, hah! Doctor! I've got it! I've got it!" when the doctor, in his calm self-possession, simply asked, "Got what, ma'am?" "Why," said she, "that snake! Now, Doctor, I told you so; I knew it; and here it is" (handing him a neat-looking paper parcel). My readers must not be surprised to learn that the contents of the parcel proved to be the same old plug of cotton inserted at the clinic, which, with the vaseline on it and the superadded viscid secretion covering it and the string all over, had, in truth, much the appearance of a member of the reptilian fraternity. The doctor, as wise and self-possessed as ever, said nothing to thwart her gratification, but allowed her free indulgence in that *conceit* which is said to be sometimes as efficacious in curing as it is frequently in killing.

The true homœopathician, on examining a case for treatment, takes into account *every symptom*—objective and subjective—not only such symptoms as seem to be in immediate connection with the special ailment he is called upon to treat, but even the most apparently remote—*the entire constitution.* He underlines those which are the most characteristic of the patient's suffering—those to which the patient gives most prominence in relating his or her ailment and those which are most noticeable by the physician. Symptoms that are common to a very large number of remedies, such as constipation, etc., he does not regard as very characteristic, except as they may be characterized by some peculiarity, for instance, "stools crumbling at the verge of the anus," "stools which recede after having been partially expelled," etc. If the symptoms be equally divided among two or more remedies, one remedy having only a part and another remedy the remainder, preference must be given to that remedy which contains the most characteristic symptoms of the case. If the most characteristic symptoms seem to be equally divided among, and equally characteristic of, two or more remedies, then some other symptoms or symptom—sometimes an apparently very remote or insignificant one—must be sought for in the pathogenesis of the respective remedies. This explains the expediency of taking the *totality of the symptoms* before deciding on the choice.

Particular attention should also be paid to the *time*, as well as to all the *conditions, of aggravation and amelioration.*

But as there are no two cases of sickness exactly alike in their most comprehensive semiology, so it will *never*—hardly ever—be found that any two medicines will be exactly alike in their respective full pathogeneses. There must be one remedy alone whose symptomatology most closely corresponds to a given case of sickness at the time of examination, and the knowledge essential to this discrimination—which, by the way, ignores the unjustifiable practice of alternation—is to be gained only by a diligent and thorough study of the MATERIA MEDICA.

The mode of examining the patient for all the symptoms is of no less importance, as taught in Hahnemann's *Organon of the Healing Art—sine qua non.*

After the exhibition of a remedy that is homœopathic to a given case, it is not uncommon to find that symptoms in the case which were not observed in the pathogenesis of the drug also disappear, leaving the patient well. These latter symptoms are properly incorporated in our materia medica, not only because they have been cured, but also because of the possibility of their development in a more extensive drug-proving. So far it is interesting as well as instructive to closely watch and verify the actions of our remedies, even our most extensively—and best—proven ones; not, however, with the vain speculations of the Milwaukee philosophers, who tried to prove (or rather to *disprove*) the well-authenticated virtues of our orthodox Aconite.

On the other hand, it frequently happens that when a homœopathic remedy is applied other symptoms which belong to the pathogenesis of the drug arise in the patient. This is more especially the case when the remedy has been administered in a high degree of attenuation.

"Guiding symptoms," "characteristics," and "key-notes" can never be substituted for the materia medica in full; but they serve the grand purpose of "guiding" us in the right direction. Indeed, it is presumable that the learned veterans who have given us these *résumés* have intended them as *guides to the study of materia medica*, rather than synopses for full decision.

Nor is a "key-note" of a remedy limited to any particular disease or class of diseases, any more than one "key-note" on a musical instrument is limited to one tune. A "key-note" of a medicine may indicate its use in a vast variety of diseases, just as one musical note may be the key-note of a vast number of melodies.

For all these grand truths—so precious to the homœopathician—we are indebted not only to the immortally honorable and honored men who laid the foundation, constructed, and bequeathed to us the great temple of Homœopathia, but also to the honored and faithful men who now live and devote their energies to the increase of the superstructure, beautifying it, adorning it, and casting their gifts into the treasury of the temple. Accordingly, we have a Homœopathy that is PROGRESSIVE (not "Latter-day Homœopathy;" for this term has reference to a "homœopathic" temple whose *foundation* has been laid in "latter days").

Progressive Homœopathy has corroborated and developed some very important facts in relation to *Analogy*, which offers itself for application, according to the following aspects:

1. *Analogy*, by symptoms which in point of location, character, appearance, conditions, time, and order agree with those in the proving.

2. *Analogy*, by similar pains and sensations, although occurring in *locations different* from those affected in the provers. (Of the many cases proving this we may cite, as a single illustration, *the constrictive, grasping sensation* around the heart found in *Cactus grandiflorus;* which sensation, when felt in *other* parts of the body, has been removed by applying the same remedy, after this phase of similarity.)

3. *Analogy*, by similar appearances of totally different pathological conditions. (Example: *Lac caninum*, a remedy discarded by the ignorant, is useful in syphilitic ulcers on the penis when there are present the *smooth, shining*, and other appearances which indicate the use of *Lac. can.* in *diphtheria.*)

4. *Analogy*, by *conditions* which alike influence totally different symptoms. (Example: Bœnninghausen records a case characterized by a thick coating of mucus which persistently gathered on the teeth of a patient and became invariably aggravated *every time he shaved;* cured by *Carbo animalis*$^{30}$, the only remedy in whose proving was found that condition of aggravation, and that, too, in connection with *a totally different symptom.* To this might be added many more examples of analogy by conditions.)

5. *Analogy* in regard to *time of aggravation and amelioration.* (We all know the value of the morning aggravations of *Nux vomica*, the 2 to 4 A. M. agg. of *Kali carb.*, the 4 to 8 P. M. agg. of *Lycopodium*, the 5 A. M. "double quick" of *Sulphur*, etc., etc.; for experience has taught us that these times of aggravation and amelioration are often reliable indications where they govern symptoms in the sick that are *entirely different* from the symptoms which furnish these indications in the proving.)

But in whatever direction we are looking for the similar remedy —whether in the direction of *pain*, of *location*, of *time*, or of *any other* aspect of analogy—we must not forget that each of these is but a direction or guide to the MATERIA MEDICA, and that we could no more expect to harmonize physiological discrepancies, by depending on a single "key-note" without the *totality of symptoms*, than we could to harmonize the monotonous repetitions of a single key-note in music without playing on the other notes of the scale.

Let us fondly cherish these deductions from pure and progressive Homœopathy. Let the living great of our noble art continue to furnish us with their experiences, and let those of us who are young in the faith be *diligent*—diligent not only in learning what we don't yet know, but also in giving our hearty co-operation to the senior workers for the furtherance of a Homœopathy that is *pure, unadulterated*, and *progressive*, that we also may, like them, be in the enjoyment of a rich experience that shall redound to the glory of Homœopathy and to the benefit of suffering humanity.

For my own part, I may say that my ignorance is fully realized when I ponder the immensity of unacquired knowledge. Like Newton—

"I feel myself playing with the shells on the shore,
While the vast ocean lies before me unexplored."

But we hope never to faint or be weary in the path of glory and duty; we feel encouraged by the veterans of our noble cause; we are still listening to the voice of SAMUEL HAHNEMANN, who "being dead, yet speaketh." He that hath ears to ear, *let him hear.*

*Auburn, N. Y., June 5th,* 1882.

